# Revascularization in Immature Permanent Teeth with Necrotic Pulp and Apical Pathology: Case Series

**DOI:** 10.1155/2017/3540159

**Published:** 2017-08-03

**Authors:** López Carmen, Mendoza Asunción, Solano Beatriz, Yáñez-Vico Rosa

**Affiliations:** ^1^Department of Pediatric Dentistry, University of Seville, Seville, Spain; ^2^Master's Program of Orthodontics, University of Seville, Seville, Spain; ^3^Department of Orthodontics, University of Seville, Seville, Spain

## Abstract

**Introduction:**

To present and discuss the results of five clinical cases treated using the revascularization protocol, showing clinical and radiographic monitoring. Necrotic immature teeth with periapical pathology present a challenge to dentists because the techniques used in apexification leave the tooth susceptible to fracture, since the root does not continue to grow in length and the canal walls are thin. Revascularization has emerged as an alternative to resolve these deficiencies, enabling apical closure, continued development of the roots, and thickening of the dentinal walls.

**Case Series:**

Five clinically and radiographically diagnosed necrotic immature permanent teeth were treated using revascularization treatment. The therapeutic protocol involved accessing the pulp chamber; irrigating copiously with NaOCl; applying a triple antibiotic paste as intracanal dressing; then provisionally sealing it. After 3 weeks, the canal was cleaned and the apex irritated with a size 15 K-file to induce blood that would serve as a scaffold for pulp revascularization. MTA was used to seal the chamber before final obturation (composite or metallic crown).

**Conclusion:**

The discussion of the results leads to debate about different restorative materials and other published protocols.

## 1. Introduction

Treating necrotic immature teeth is challenging because the thin dentinal walls and short roots make them more susceptible to fracture. The treatment of choice is apexification, which consists of inducing apical closure by using calcium hydroxide or mineral trioxide aggregate (MTA) [1, 2]. However, while apexification resolves the problem of apical periodontitis and induces apical closure, enabling definitive root canal therapy to be performed, it does not thicken the root walls or allow the root to continue to develop, leaving the tooth susceptible to fracture [3].

The protocols referred to as regenerative endodontic procedures are currently being revived as a new paradigm in conservative treatment. It was previously thought that regenerating the necrotic pulp of an immature tooth with periapical periodontitis was impossible; nowadays however we know that, in a suitable medium, this can be achieved using the technique known variously as “revascularization” [4, 5] or “revitalization” [6].

Proper disinfection of the canal, a suitable matrix for new tissue ingrowth, and an effective seal for the coronal access are vital for a satisfactory result [7], without forgetting the importance of a supply of dental stem cells so that revascularization can be successfully completed [2, 8]. So many articles have reported satisfactory results in necrotic immature teeth as a result of the revascularization technique, that the uncertainty that arose some years ago about whether it was possible to determine the success of this treatment has now been dispelled. The new question now is which protocol should be followed? The objective of this study is to present a series of cases, as well as gather information about revascularization, the technique, and results.

## 2. Case Series

### 2.1. Case  1

An 8-year-old patient went to the pediatric dental clinic with symptoms suggesting acute pulpitis, with a sharp pain that increased at night and did not ease with analgesics. No abscess was observed upon clinical examination, although there was a large cavity. On periodontal examination, no pathologic tooth mobility was observed, and probing depth was less than 3 mm.

**Figure 1:**
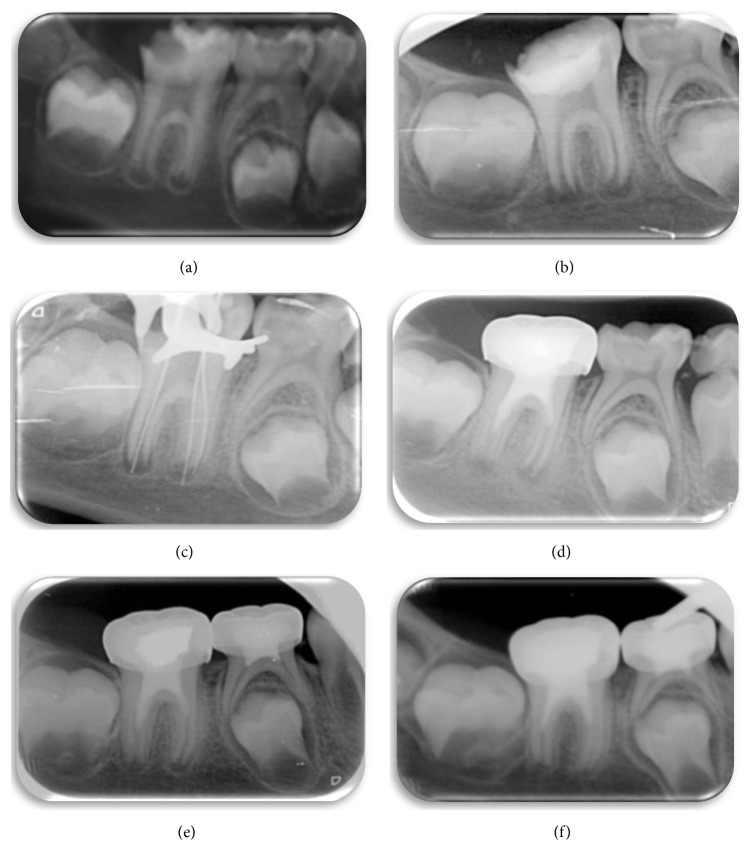
(a) Initial radiograph of large occlusodistal caries on tooth 36, extending to the pulp chamber and with periapical translucencies. (b) Radiograph showing triple antibiotic paste in the canal. (c) A size 15 K-file is used to irritate the apical tissue to induce bleeding. (d) Periapical radiograph of MTA over the blood clot and the pulp chamber sealed with glass ionomer, composite, and metal crown. (e) Follow-up radiograph taken 6 months after completion of treatment. By comparing this radiograph with those taken previously, the periapical radiotranslucency has decreased and there is further closure of the apical foramen. (f) Follow-up of 12 months after carrying out revascularization treatment. The periapical radiotranslucency has practically disappeared; there is further thickening of the dentinal walls and the apical foramen has almost closed.

Radiographically, a mesioocclusodistal caries was observed on tooth 46, extending into the pulp chamber. The image showed periapical radiotranslucency and open apices. The patient was prescribed preliminary antibiotic treatment (Amoxicillin 50 mg/kg) (Figure 1(a)).

After evaluating the therapeutic options, we opted for revascularization treatment. Under local anesthesia and using a rubber dam, the crown was cleaned with 2% chlorhexidine. The pulp chamber was accessed and the canal irrigated with 5% NaOCl. Working length was determined with size 13 K-files, minimal instrumentation and constant irrigation. Finally, the triple antibiotic paste (metronidazole, ciprofloxacin, and minocycline) was placed in the canal in equal proportions, mixed with sterile water, and the chamber was temporarily sealed with IRM and glass ionomer cement (Figure 1(b)).

After 3 weeks, the paste was removed with copious irrigation of 5% NaOCl. Then, a file was inserted into the root to induce apical bleeding, allowing 15 minutes for the clot to form. Some 2 mm of MTA was placed in the chamber part of the canal and covered by a wet cotton pellet for an hour, before final obturation with glass ionomer cement, composite, and metal crown (Figure 1(c)). At 6 and 12 months, the periapical pathology has noticeably improved, then healed, and increased dentinal wall thickness and an almost closed apical foramen can be observed (Figure 1(d)).

### 2.2. Case  2

An eight-and-a-half-year-old patient went to the pediatric dental clinic six months after a trauma that caused subluxation of tooth 21. At the clinical examination, change of color was observed in the crown of 21 and a periapical abscess had appeared. There was no response to the cold tests to determine pulp vitality, whereas there was a response in the contralateral teeth. No pathologic mobility was observed at the periodontal examination. This led us to consider pulp necrosis.

**Figure 2:**
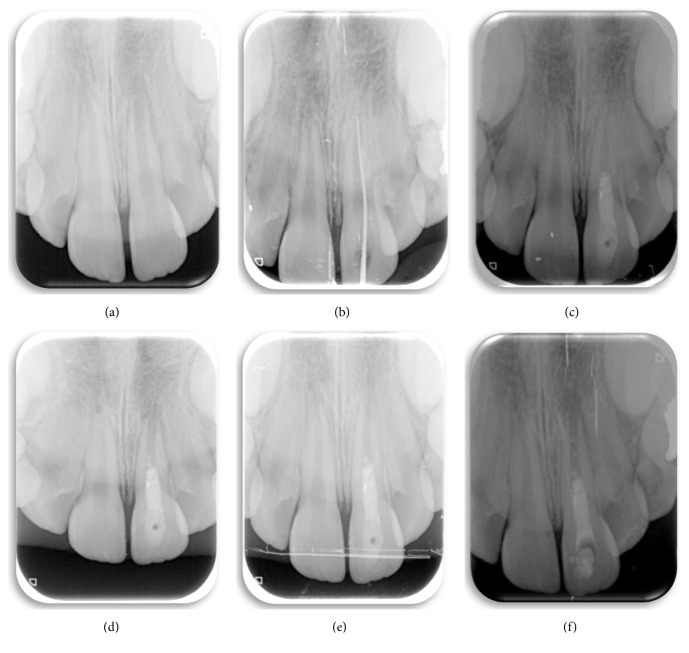
(a) Periapical radiograph image, 6 months after a trauma-induced subluxation of tooth 21, shows open apex of more than 2 mm, with periapical radiotranslucency. (b) Start of revascularization treatment of tooth 21, determination of working length. (c) Chamber sealed with MTA and wet cotton pellets, waiting for it to set hard. (d) Follow-up radiograph at 3 months, the periapical radiotranslucency at tooth 21 has disappeared. (e) Seven months after completion of revascularization therapy, dentinal wall thickening can be observed at 21, with the consequent narrowing of the apical foramen. (f) One year and 7 months after completion of revascularization treatment and compared with the periapical radiograph taken when revascularization treatment ended, there is a noticeable increase in root length, similar to the adjacent central incisor, thickening of the root walls, and a closed apex.

If we radiographically compare the roots of the two central incisors, we can see that the apex of 21 is considerably less developed than that of 11, and tooth 21 is between Nolla's developmental stages 8 and 9. The image showed periapical radiotranslucency. The patient received preliminary antibiotic treatment (Amoxicillin 50 mg/kg) (Figure 2(a)).

Revascularization treatment was performed following the same protocol as before (Figures 2(b) and 2(c)). At the control visits, 3 and 7 months after completion of revascularization treatment, the radiotranslucency at 21 has disappeared, and thickening of the dentinal walls and the consequent narrowing of the apical foramen can also be observed (Figures 2(d) and 2(e)). One year and 7 months after revascularization, there is an appreciable increase in root length and root wall thickening and the apex has closed (Figure 2(f)).

### 2.3. Case  3

**Figure 3:**
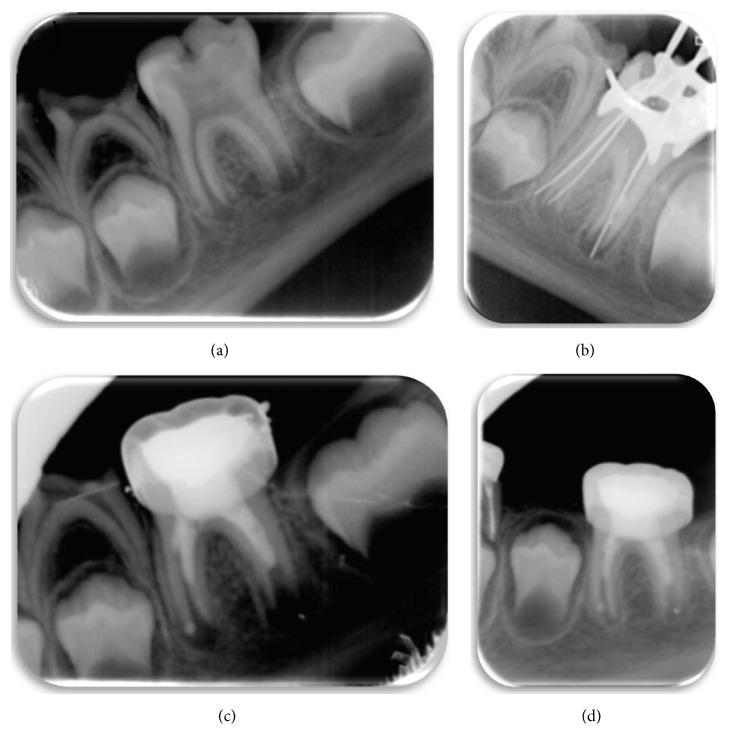
(a) Initial periapical radiograph: occlusal caries can be observed extending into the pulp chamber and radiotranslucency on the root apices. (b) A size 15 K-file is used to irritate the apical tissue and stimulate bleeding. (c) Completion of revascularization. (d) Follow-up periapical radiograph, 8 months after completion of revascularization. The periapical radiotranslucency has disappeared and the walls of the root canal have thickened; apical closure has still not taken place. This should occur at the following check-up.

A six-and-a half-year-old patient went to the pediatric dental clinic with multiple caries. Tooth 36 presented a large area of occlusal caries, and the patient experienced acute pain, increasing at night. The radiograph showed occlusal caries on tooth 36, extending into the pulp chamber. There were periapical radiotranslucency and open apices. Preliminary treatment with antibiotics was prescribed (Amoxicillin 50 mg/kg) (Figure 3(a)). Revascularization treatment was carried out, following the same procedure as above (Figures 3(b) and 3(c)). After 8 months, the periapical radiotranslucency has disappeared and the walls of the root canal are thicker. Apical closure has not yet taken place, although it is expected to have occurred by the next check-up (Figure 3(d))

### 2.4. Case  4

**Figure 4:**
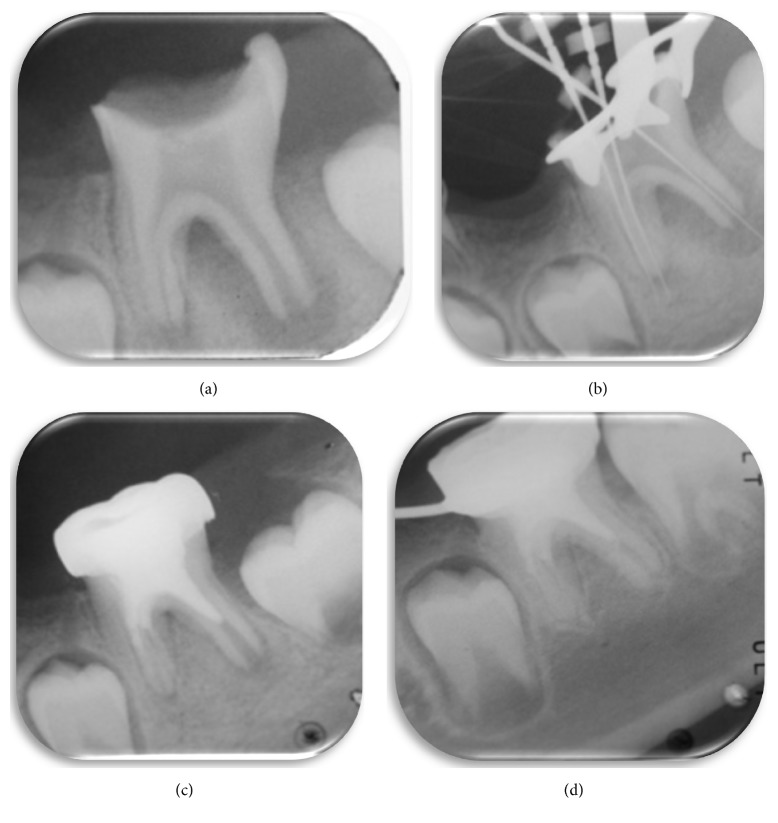
(a) Periapical radiograph taken at the first visit, showing large occlusal caries on tooth 36 with pulp exposure, open apices, and a radiotranslucency encompassing the furcation and the distal root of the molar. (b) Size 15 K-files are used for apical irritation to induce bleeding. (c) Completion of revascularization treatment after placing the MTA and metal crown. (d) Check-up at 9 months. The radiotranslucency has completely disappeared. Obliteration of the two mesial canals can be observed.

An eight-year-old patient went to the pediatric dental clinic with multiple caries. Tooth 36 presented occlusal caries extending into the pulp chamber and open apices, with a large radiotranslucency covering the whole of the distal root surface and the molar furcation (Figure 4(a)). The prognosis for this molar was negative, with revascularization treatment being the final option to try and save it, following the same protocol as described above (Figures 4(b) and 4(c)). Preliminary antibiotic treatment was prescribed (Amoxicillin 50 mg/kg).

A check-up was carried out at 9 months, when it was observed that the radiotranslucency on the root and round the furcation had disappeared and the walls of the root canal had thickened. The apex of the distal root had not yet completely closed, although the apex of the mesial root had. Obliteration of the mesial canals can also be observed (Figure 4(d)).

**Figure 5:**
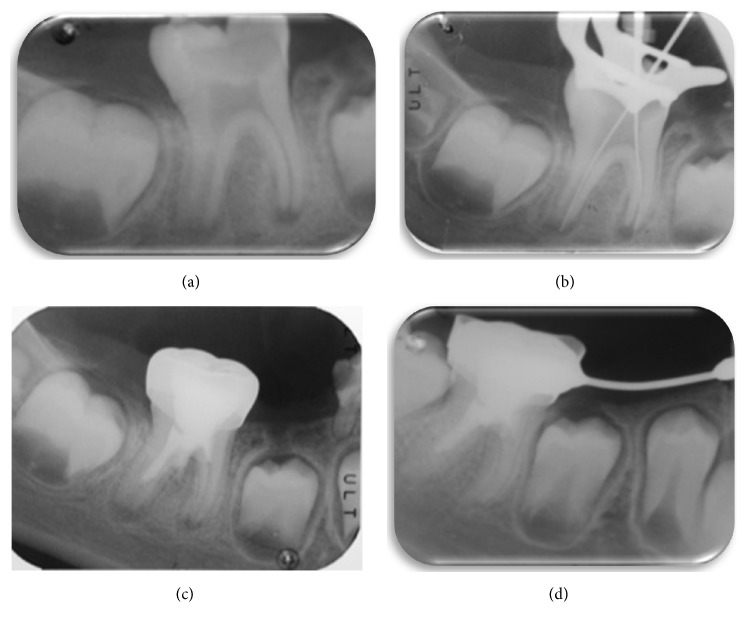
(a) First visit showing occlusal caries on tooth 46 extending to the pulp chamber, with open apices. (b) Determination of working length. (c) End of revascularization therapy, having placed MTA and metal crown. (d) Follow-up radiograph. Apical closure of both roots is shown and radiotranslucencies have disappeared. A complication associated with revascularization is obliteration of the root canals that is shown here.

In addition, 46 presented occlusal caries extending into the pulp chamber and open apices (Figure 5(a)). Revascularization treatment was performed, following the protocol described (Figures 5(b) and 5(c)). Preliminary antibiotic treatment was prescribed (Amoxicillin 50 mg/kg). After 9 months, the periapical radiolucency had disappeared, the walls of the canal had thickened, and the root apices were fully closed. As a complication of revascularization, the obliteration of all its root canals is evident. (Figure 5(d)).

## 3. Discussion

The procedure to be followed for revascularization treatment in necrotic immature permanent teeth is not standardized [9] and there is considerable controversy to date. Nonetheless, substantial number of authors seem to support the following protocol: first, a preoperative radiographic evaluation should be carried out to determine the degree of root development and the periapical status of the tooth. After local anesthesia, the tooth is isolated with a rubber dam and the crown cleaned with 2% chlorhexidine. Next, the pulp chamber is accessed and the root canals are irrigated copiously in order to disinfect them.

Given the lack of a standardized protocol for revascularization, Trevino et al. [10] investigated the possible harmful effect that different irrigants may have on stem cells from the apical papilla (SCAP). They concluded that 17% EDTA promotes SCAP survival and allows them to become attached to the root canal dentinal wall, so that its use in an irrigation protocol could be very beneficial for revascularization. Irrigation with 2% chlorhexidine on the other hand was detrimental to SCAP viability [10]. Using histologic observations from animal testing, da Silva et al. [11] obtained promising results on teeth with apical periodontitis using the negative pressure irrigation system, EndoVac, to disinfect the root canal. The authors suggest that this technique makes the application of intracanal antibiotics unnecessary.

In all the cases described, minimal instrumentation was used in the root canals to ensure that the infected pulp tissue was eliminated but without weakening the canals. In accordance with the research of Jadhav et al. [12] and Chen et al. [5] involving humans, as well as others who used an animal model [11], we decided to use gentle instrumentation for the reason mentioned above. Other authors, however, suggest that no instrumentation should be used on animals [13, 14] or humans [15]. After irrigating again with 5% sodium hypochlorite, the canals were dried with absorbent paper points, before proceeding to disinfection with intracanal medication, since all the literature coincides in stating that NaOCl is rarely sufficient for successful disinfection in revascularization [3, 5, 8, 11, 13–15]. Nevertheless, current protocols might be more conservative in terms of stem cells damage, so 17% EDTA might be sufficient for successful irrigation.

The intracanal medication should be used at concentrations that are bactericidal but do not simultaneously prejudice stem cell viability [6]. The literature describes the use of Ca(OH)_2_ [3, 5, 15] or a paste consisting of three antibiotics [8, 11, 13, 14].

Few of the published studies used intracanal Ca(OH)_2_, rather than the triple antibiotic paste, although all of them achieved good results [3, 5, 15].

Bose et al. [3] observed that although the different medications produced apical closure and increased root length, a greater increase in dentinal wall thickness was observed with the triple antibiotic paste. With respect to the triple antibiotic paste, most authors advocate using equal proportions of ciprofloxacin, metronidazole, and minocycline, mixed with sterile water, since the flora in the root canal system is polymicrobial. A mixture of these three antibiotics has shown satisfactory results for disinfecting the canals and the healing of periapical lesions [8, 11, 13, 14].

The use of this paste is not without its complications, since it can lead to discoloration of the tooth crown, the development of bacterial resistance, and the appearance of allergic reactions [2, 11]. Moreover we should be aware that root resorption and active apical periodontitis lesion might occur when following these protocols [13–17]. In a comparative study concerning the components of the triple antibiotic paste, only minocycline caused tooth discoloration, so that it is recommended to restrict the use of this paste to the root canal and not to go beyond the cementoenamel junction [17]. In three of the cases presented, 1, 3, and 4, we were unable to evaluate discoloration since the molars had been fitted with metal crowns. In case 2, the patient arrived at the clinic with discoloration of tooth 21 before revascularization treatment started, due to a trauma 6 months previously. After completion of treatment, however, an increase in the change of color to gray was observed.

The mean period of time considered necessary for the bacterial disinfection of the canals, leaving the triple antibiotic paste in until they are clean, varies between 1 and 4 weeks, depending on the author. While Ding et al. [4] (in their clinical study with humans) suggest waiting for 1 week, other authors, in experimental research, suggest 2 weeks [11, 13], others, along with us, advocate leaving the triple antibiotic paste for 3 weeks [15], and some have even proposed that it be left for four weeks [5].

Inducing intracanal bleeding plays an important role in the success of this therapy. Using a file to irritate the apex, the fundamental reason is to cause the formation of a blood clot in the canal space, which then serves as a scaffold to enable the three-dimensional ingrowth of new tissue [18]. There is a sort of agreement about the importance of stimulating apical bleeding to form the clot that will then act as a scaffold for pulp revascularization [4, 5, 12–15, 18, 19].

In case 4, the follow-up radiographs showed obliteration of some of the canal spaces. The literature describes partial or complete obliteration of the pulp canal as one of the complications of revascularization in necrotic immature teeth, although the exact mechanism for the calcification has not been found [5]. The formation of cementum bridges may be due to the use of MTA, which has osteoinductive capacity [19, 20]. The presence of calcification in the canal may prejudice the esthetics, as well as create difficulties for any future endodontic treatment that may be necessary [21]. Moreover, this type of treatment does not represent an immediate solution, which in current days seems to be the most demanded type of treatments by our patients, based on the unability to delay gratification behavior widely spread in our society and current children [22]. Despite all the aforementioned, the technique referred to as regenerative endodontics, revascularization, or revitalization is therefore an effective and conservative option for necrotic immature teeth. In addition to promoting apical closure, it permits further root lengthening and thickening of dentinal walls, so resolving the problem of susceptibility to fracture that used to appear following apexification therapy. However, there are still too few large-scale clinical studies involving humans and more research is needed.

## 4. Conclusion

1. For necrotic immature teeth, revascularization is a desirable alternative to apexification and shows both good short-term and long-term prognosis [17].
2. A positive response to the pulp vitality test may appear after a year; this is an encouraging result, which indicates that this technique is preferable to apexification [4]. Nonetheless, more randomized clinical trials using a greater number of samples are needed.
3. Although the general trend is to use different concentrations of NaOCl as an irrigant, 17% EDTA seems to have very interesting properties that favor SCAP survival and, therefore, revascularization.
4. For the future, more studies are needed to improve the results of regenerative endodontics therapy in immature teeth with pulp necrosis.
